# High double burden of child stunting and maternal overweight in the Republic of the Marshall Islands

**DOI:** 10.1111/mcn.12832

**Published:** 2020-08-24

**Authors:** Jessica L. Blankenship, Stanley Gwavuya, Uma Palaniappan, Julia Alfred, Frederick deBrum, Wendy Erasmus

**Affiliations:** ^1^ UNICEF East Asia and Pacific Regional Office Bangkok Thailand; ^2^ UNICEF Pacific Office Suva Fiji; ^3^ Ministry of Health Majuro Republic of the Marshall Islands; ^4^ Economic, Policy, Planning and Statistics Office Majuro Republic of the Marshall Islands

**Keywords:** children, double burden of malnutrition, maternal obesity, Pacific, Republic of the Marshall Islands, stunting

## Abstract

Many low‐ and middle‐income countries are faced with a double burden of malnutrition characterized by a stagnating burden of undernutrition and an increasing prevalence of overweight and obesity often observed both at population and household levels. We used data from the 2017 National Integrated Child Health and Nutrition Survey in the Republic of the Marshall Islands to explore the prevalence of overweight mother‐stunted child pairs (mother–child double burden, MCDB). We used bivariate analysis, multivariate logistic regression, and multinomial logistic regression analysis to explore associations between child‐, maternal‐, and household‐level variables and both stunting and MCDB and other types of maternal–child pairs. Our results indicate that nearly three out of four mothers were overweight or obese and one in four households is home to an overweight mother with a stunted child. The risk of child stunting and of MCDB were largely associated with maternal characteristics of lower maternal height, maternal age at birth, years of education, and marital status and household economic status as measured by wealth index and number of household members. These findings support the growing body of evidence showing that the coexistence of high maternal overweight and child stunting (MCDB) has linked root causes to early life undernutrition that are exacerbated by the nutrition transition.

## 1 BACKGROUND

Many low‐ and middle‐income countries are faced with a double burden of malnutrition characterized by a stagnating burden of undernutrition and an increasing burden of overweight and obesity (Gillespie & Haddad, 2001). This phenomenon can occur at the population level, within communities and households, and even in the same person. Globally, the double burden of malnutrition is largely attributed to the nutrition transition in which populations shift from consumption of traditional nutrient‐dense and low‐fat diets to a “Western” diet consisting of high‐energy, nutrient‐poor foods (Ghattas, 2014; Popkin, 2002; Ziaullah, 2014). These processed convenience and snack foods are increasingly available and affordable in low‐ and middle‐income countries as a result of economic growth and urbanization (Food and Agriculture Organization [FAO], 2006; Hoffman, 2001).

Discordance in nutrition status of mother–child pairs is a more extreme form of the double burden of malnutrition. It is commonly assumed that mothers and their children should be less likely to differ in their nutrition status as they have shared access to food, sanitation, hygiene, and other resources. However, in low‐ and middle‐income countries, the nutrition transition, along with increasingly sedentary lifestyles, has changed eating and activity patterns in both children and their mothers with increased consumption of energy dense and nutrient poor foods for all members of the household. These foods contribute to increased overweight in adults and fail to help young children meet their linear growth potential (Tzioumis & Adair, 2014). The mother–child double burden (MCDB) of malnutrition is an established concern globally. In a systematic review of the prevalence of the mother–child double burden in 50 countries, the prevalence was less than 10% for nearly all countries with the exception of Guatemala (20%), Egypt (16%), and Bolivia (11.5%; Kosaka & Umezaki, 2017).

MCDB has linked root causes to early life undernutrition that are further exacerbated by the nutrition transition (Delisle, 2008; Popkin, Adair, & Ng, 2012; Gillespie & Haddad, 2001). Increased risk of MCDB is associated with increasing urbanization (Food and Agriculture Organization [FAO], 2006), socio‐economic status (Doak CM., 2005; Jehn M., 2009; Lee J., 2010), household food insecurity (Gubert MB., 2016; Ghattas, 2014), and poor dietary quality (Delisle, 2008). Maternal short stature, indicative of early undernutrition of the mother, is strongly associated to increased risk of child stunting (Garrett J.L., 2005), maternal overweight (Sichierei R., 2003; Bosy Westphal A., 2009), and of MCDB (Ramirex‐Zea M., 2014; Oddo VM., 2012). There are several proposed mechanisms explaining the link between early childhood stunting and later overweight and obesity in adulthood. Children stunted during the first 1,000 days are likely to undergo metabolic and physiological changes in response to undernourishment, including impaired fat metabolism (Frisancho 2003, Friedman SM 2006, Hoffman, Sawaya, et al., 2000), accumulation of excess body fat (Sawaya, 2003; Sawaya, Grillo, Verreschi, Carlos da Silva, & Roberts, 1998), higher energy intake for body weight (Hoffman, Roberts, et al., 2000), lower resting metabolic rate (Grillo LP. 2005), and lower energy (Hoffman, Sawaya, et al., 2000, Friedman SM 2006). In obesogenic environments, these metabolic and physiological adaptations for energy and fat conservation can lead to overweight and increase susceptibility to develop noncommunicable diseases (NCDs) later in childhood and into adolescence and adulthood (Hoffman, Roberts, et al., 2000; Sawaya, 2003; Sawaya et al., 1998).

Existing research on the MCDB is focused on countries in South America, Asia, and Africa with no research available from Pacific Island Nations despite the region having the highest prevalence of child stunting (Helble & Francisco, 2017) concurrent with the highest prevalence (~60%) of overweight adults in the world (World Health Organization, 2016). The recent national Integrated Child Health and Nutrition Survey (Republic of the Marshall Islands Ministry of Health and Human Services, 2017) in The Republic of the Marshall Islands (RMI) confirmed a high prevalence of MCDB in RMI with 25% of household with children under 5 years of age containing both a stunted child and an overweight or obese mother. This prevalence places RMI with the highest recorded prevalence of MCDB in the world.

This analysis examines the prevalence of MCDB in RMI and explores associated child, maternal, and household variables that influence the risk of child stunting alone and of MCDB compared with other types of maternal–child pairs. As urbanization and economic growth continue to progress in RMI, identification of households at risk for MCDB and an understanding of the influencing variables will guide policy makers to best address the growing divide of malnutrition in the country.

## 2 METHODS

### 2.1 Data

We utilized nationally representative data for households with children under 5 years from the 2017 RMI ICHNS conducted by the RMI Ministry of Health and Human Services and Economic Policy, Planning and Statistics Office. A detailed description of the survey design and sample selection can be found elsewhere (Republic of the Marshall Islands Ministry of Health and Human Services, 2017). Briefly, the sample was based on the 2011 RMI census with 2017 projections used as the sampling from which a two‐stage, stratified cluster sampling approach was used to for the selection of the survey sample. A total of 50 clusters (communities) stratified for urban and rural areas were selected using probability proportion to size on all islands in RMI with a population over 100 people. In each selected cluster, a household listing was carried out to identify household with children under 5 years of age and 12 households in each of the 50 clusters were randomly sampled as the secondary sampling unit for a total of 600 children. Several clusters had less than 12 households with children under 5 years of age necessitating a reduction in the total sample size to 581 households. Household eligibility was based on a child 0–59 months of age living in the household. The RMI ICHNS 2017 was not self‐weighting with sample weights calculated to account for the probability of a household and child selection based on the 2010 Census and the level of nonresponse to the survey. The survey consisted of a household questionnaire and questionnaires for mothers and children <5 years, which were based on the UNICEF Multiple Indicator Cluster Survey (MICS) 6 and 2007 RMI Demographic and Health Survey (DHS; Economic Policy, 2007).

### 2.2 Key variables

Child, maternal, and household characteristics included in the analysis were selected based on the UNICEF conceptual framework for the determinants of child undernutrition (UNICEF, 2013) and on previous examinations on drivers of MCDB (Oddo et al. 2012, Kosaka & Umezaki, 2017). The nutritional status of children and their mothers was assessed using height and weight data in accordance with the WHO 2006 Child Growth Standards (WHO MultiCentre Growth Reference Study Group and de Onis, 2006) and calculation of maternal body mass index (BMI; kg/m^2^), respectively. Children <5 were classified as stunted when their height‐for‐age *Z*‐score < −2 *SD* and mothers with a BMI ≥ 25 kg/m ^2^ or BMI ≥ 30 kg/m ^2^ were classified as overweight and obese respectively. MCDB was defined by four categories: (a) stunted child–overweight mother pairs (corresponding to MCDB pairs), (b) stunted child–nonoverweight mother pairs, (c) nonstunted child–overweight mother pairs, and (d) nonstunted child–nonoverweight mother pairs.

Potential covariates for child undernutrition were examined at the child, maternal, and household levels. Variables collected for children included child sex, child age (recoded as a dummy variable to allow for nonlinear associations), incidence of diarrhoea in the previous 2 weeks, and being left in inadequate care in the previous week. Mothers' characteristics included current height as a proxy for prior stunting experience, marital status, level of schooling, and age at child's birth. Maternal height was defined by three categories: maternal height <150 cm, ≥150 cm and ≤159.9 cm and ≥160 cm (Ozaltin, Hill, & Subramanian, 2010).

Household‐level variables included geographic area, wealth index quintile, practice of open defecation, and access to handwashing facilities inside the house. Geographic area was divided into two categories: rural and urban, as identified by the RMI 2011 census. Wealth index quintiles were calculated using principle component analysis of household characteristics from the national sample (Fry, Firestone, & Chakraborty, 2014). Classification of open defecation and access to handwashing facilities inside the house were assessed using standardized methodology (WHO and UNICEF, 2014).

### 2.3 Statistical methods

Bivariate analyses were conducted to determine the association between known determinants of child undernutrition (UNICEF, 2013) with stunting and MCDB. For MCDB, multinomial logistic regression was conducted to determine the associated risk of MCDB with influencing variables from the child, maternal, and household levels compared with other types of maternal–child nutrition status pairs. Results are expressed as relative risk ratios (RR) with 95% confidence intervals. For child stunting, binomial logistic regression was conducted to determine the associated odds of stunting with predicting variables from the child, maternal, and household levels. Results are expressed as adjusted odds ratios (AORs) with 95% confidence intervals. A *p*‐value of less than.05 considered statistically significant for both stunting and MCDB models. Factors were included in the multivariate and multinomial logistic regression models based on a significant (*p* < .05) bivariate relationship and/or evidence‐based factors associated with children's nutritional status. Sampling weights were utilized to account for the unequal probability of selection resulting from the two‐stage cluster randomized sample design and nonresponse. Statistical analyses were conducted using SPSS version 22.0 (Armonk, NY: IBM Corp) software using the complex samples module.

## 3 RESULTS

### 3.1 Sample characteristics

A total of 581 households with 881 children <5 years were included in the survey with 100% of all sampled children and their caregivers participating in the survey. About 858 (97.4%) of the children included in the survey had complete height and weight data, 16 were excluded due to the presence of oedema, 6 were excluded due to implausible height‐for‐age *Z*‐score or weight‐for‐height *Z*‐score scores (WHO MultiCentre Growth Reference Study Group and de Onis, 2006), and 4 were excluded due to missing data. An additional 129 children were removed from the analysis as they did not live with their mother. There were no significant differences in the prevalence of stunting, child sex, area, or wealth index quintile between these children, and 729 children (464 households) were included in the analysis (Table 1).

**Table 1:** Socio‐demographic and anthropometric characteristics of the sample population

Survey population characteristics	Mean ± *SD* a
*Child characteristics n = 729*	
Age (months)	29.4 ± 17.3
Sex (% male)	51.7
Diarrhoea	10.1
Left in inadequate care	8.9
*Maternal characteristics* b *n* = 576	
Age at birth (years)	27.1 ± 6.3
Education in years	10.8 ± 3.1
Mother never married or cohabitating	44.5
BMI (kg/m^2^)	29.1 ± 6.3
Height (cm)	1.52 ± 0.5
Height categories	
<150 cm	27.9
150–159 cm	65.8
≥160 cm (reference)	6.3
Nutrition status	
Underweight (BMI < 18.5)	2.0
Normal (BMI 18.5–24.9)	27.5
Overweight (BMI ≥ 25.0)	28.4
Obese (BMI ≥ 30.0)	42.1
*Household characteristics n = 464*	
Wealth index quintile^c^	
Poorest	21.7
Poorer	22.9
Middle	21.3
Richer	16.9
Richest	17.2
Area (% urban)	81.1
Number of household members	9.1 ± 4.7
Handwashing facility in the household	30.7
Open defecation	11.2

Abbreviation: BMI, body mass index.

a Percentages presented where appropriate.

b Only mothers of sampled children were included in analysis with other caregivers excluded.

c Wealth index quintiles were developed using principal component analysis summarizing housing variables (e.g., roof, walls, and floor) and asset variables (e.g., television, radio, and car).

The majority (81.1%) of households were located in urban areas, with 44.6% and 34.1% of households in the two lowest and two highest national wealth index quintiles, respectively (Table 1). The average household size comprised 9.1 members. Boys (51.7%) and girls (48.3%) were equally represented. Mothers' mean age, BMI, and height were 27.1 years, 29.1 kg/m^2^, and 152 cm, respectively. Mothers had a mean number of 10.8 years of education, and 44.5% of them were either not married or cohabitating with a partner. The prevalence of overweight and obesity among mothers was high: 70.5% of mothers were either overweight or obese (28.4% with BMI ≥25 and <30 and 42.1% with a BMI ≥30).

More than one‐third (37.1%) of children were stunted with the highest prevalence of stunting (60.0%) among children 18–23 months of age (Table 2). The prevalence of wasting and overweight among children was low (4.0% and 3.3% respectively). Among maternal–child pairs, 25.2% were MCDB with an overweight or obese mother and a stunted child (Table 3).

**Table 2:** Nutritional status by child age

Children 0‐59m	Stunted children**		Wasted		Overweight*	
Age category	(%)^a^	n	(%)^b^	n	(%)^c^	*n*
0–5	14.9	71	4.6	70	6.2	70
6–11	21.7	92	5.7	88	3.4	88
12–17	35.9	68	8.1	67	6.5	67
18–23	60.0	69	1.5	68	8.8	68
24–35	45.8	149	5.0	147	3.5	147
36–47	33.6	136	2.2	132	0.0	132
48–59	41.2	144	2.8	142	0.7	142
Total	37.1	729	4.0	714	3.3	714

a Stunted defined as height‐for‐age *Z*‐score (HAZ) < −2 *SD*.

b Wasted defined as weight‐for‐height *Z*‐score (WHZ) < −2 *SD*.

c Overweight defined as weight‐for‐height *Z*‐score (WHZ) > +2 *SD*.

* *p* < .01.

** *p* < .001.

**Table 3:** Total prevalence of double burden of malnutrition in households

Children 0‐59m	Maternal nutrition status (%)				
Child nutrition status (%)	Underweight (BMI < 18.5)	Normal (BMI 18.5–24.9)	Overweight (BMI 25–29.9)	Obese (BMI ≥ 30)	Total overweight/obese (BMI ≥ 25)
Stunted (height‐for‐age < −2)	0.4	11.4	11.5	13.7	25.2
Underweight (weight‐for‐age < −2)	0.3	4.6	3.6	3.3	6.9
Wasted (weight‐for‐height < −2)	0.3	1.4	1.7	0.6	2.3
Overweight/obese (weight‐for‐height > +2)	0.0	0.9	0.9	1.4	2.3

*Note. n* = 729.

Abbreviation: BMI, body mass index.

### 3.2 Influencing factors for stunting

Variables associated with increased odds of stunting in children include older age of the child, larger number of household members, lower household wealth index, shorter maternal height, and unmarried or cohabitating maternal status (Table 4). Compared with mothers taller than 160 cm, mothers who were shorter than 150 cm had a 6.6 greater odds of having a stunted child (AOR = 6.62; 95% CI = 2.50, 17.51), and mothers whose height was 150–159 cm tall had a 4.5 greater likelihood of having a stunted child (AOR = 4.55; 95% CI = 1.66, 12.50). Mothers who were never married or cohabitating were more likely to have a stunted child compared with married mothers (AOR = 1.72; 95% CI = 1.07, 2.77). Children from lower wealth quintiles were more likely to be stunted compared with children from the wealthiest quintile; the highest odds of stunting were observed among children from the poorest wealth quintile (AOR = 3.13; 95% CI = 1.54, 6.37).

**Table 4:** Associations of child, maternal, and household influencing variables of stunting in children 0–59 months of age and with mother–child double burden pairs^a^

Population indicators	Stunted^b^	Mother child double burden (MCDB) in comparison to:		
		Non‐stunted child nonoverweight mother^c^	Stunted child nonoverweight mother^d^	Nonstunted child overweight mother^e^
	AOR (95% CI)	RR (95%CI)		
Child characteristics				
Age (months)				
0–5 m (reference)				
6–11 m	1.58 (0.47–5.28)	2.27 (0.64–7.98)	0.53 (0.11–2.64)	0.87 (0.28–2.70)
12–17 m	3.87 (1.14–11.93)**	3.76 (1.02–14.02)*	0.63 (0.12–3.18)	2.58 (0.80–8.33)
18–23 m	10.67 (3.83–29.69)***	13.46 (3.76–48.15)***	1.37 (0.30–6.28)	10.87 (3.53–33.50)***
24–35 m	6.25 (2.60–15.04)***	34.19 (10.45–111.87)***	3.61 (0.83–15.74)	5.1 (1.87–13.88)**
36–47 m	3.42 (1.41–8.34)**	8.19 (2.66–25.27)***	2.13 (0.48–9.40)	3.13 (1.14–8.57) *
48–59 m	4.75 (1.76–12.81)***	13.60 (4.28–43.26)***	1.73 (0.41–7.35)	3.63 (1.33–9.91)*
Male child	1.58 (0.95–2.622)	1.41 (0.82–2.40)	1.06 (0.60–1.86)	1.59 (1.06–2.39)*
Child left in inadequate care	0.94 (0.46–1.93)	0.39 (0.15–1.02)	0.53 (0.20–1.41)	0.99 (0.47–2.09)
Diarrhoea	0.87 (0.47–1.63)	0.32 (0.14–0.75)	0.57 (0.23–1.41)	1.08 (0.52–2.22)
Maternal characteristics				
Age at birth (years)	1.00 (0.97–1.04)	1.19 (1.12–1.26)***	1.23 (1.15–1.32)***	1.03 (0.99–1.07)
Higher education	0.56 (0.29–1.09)	3.23 (1.28–8.33)*	4.76 (1.32–16.67)*	0.61 (0.34–1.08)
Never married or cohabitating	1.72 (1.07–2.77)**	1.30 (0.74–2.28)	1.45 (0.81–2.61)	2.32 (1.50–3.54)***
Parity	1.13 (0.94–1.35)	1.07 (0.85–1.36)	0.71 (0.56–0.90)**	1.03 (0.99–1.07)
Maternal height (cm)				
<150 cm	6.62 (2.50, 17.51)***	4.44 (1.30–15.18)*	0.60 (0.07–4.92)	6.73 (2.44–18.56)***
150–159 cm	4.55 (1.66–12.50)**	3.36 (1.03–10.99)*	0.46 (0.06–3.67)	3.73 (1.40–9.96)**
≥160 cm (reference)				
Family characteristics				
Wealth index quintile				
Poorest	3.13 (1.54–6.37)**	3.31 (0.83–12.26)	3.04 (0.75–12.30)	4.37 (1.67–11.49)**
Poorer	2.80 (1.07–7.30)**	0.92 (0.34–2.51)	1.02 (0.34–2.30)	3.96 (1.87–8.41)***
Middle	2.36 (1.34–4.14)**	2.56 (0.95–6.94)	2.16 (0.74–6.31)	3.04 (1.49–6.19)**
Richer	1.89 (1.13–3.16)*	0.68 (0.24–1.92)	1.02 (0.32–3.25)	2.27 (1.06–4.88)*
Richest (reference)				
Urban area	0.73 (0.44–1.21)	2.87 (0.94–8.72)	2.87 (0.92–8.94)	1.60 (0.70–3.64)
Open defecation	1.31 (0.61–2.84)	3.41 (1.20–9.68)*	2.53 (0.88–7.25)	1.37 (0.67–2.81)
Number of household members	1.04 (1.00–1.08)**	1.19 (1.12–1.11)**	1.01 (0.97–1.06)	1.04 (1.01–1.08)**
Handwashing place not in house	1.02 (0.54–1.91)	1.13 (0.59–2.17)	0.45 (0.21–0.95)*	0.75 (0.46–1.23)

*Note. n* = 729.

Abbreviations: AOR, adjusted odds ratio; BMI, body mass index; MCDB, mother–child double burden; RR, risk ratios.

a Multivariate logistic regression performed with baseline comparison group as children who were not stunted. Multinomial logistic regression performed with MCDB pairs and baseline comparison groups of (a) nonstunted child nonoverweight mother, (b) stunted child non‐overweight mother, and (c) nonstunted child overweight mother. Results are presented as AOR with 95% CI. All estimations are adjusted for two‐stage cluster randomized survey design and nonresponse.

b Stunted defined as height‐for‐age *Z*‐score (HAZ) < −2 *SD* below median.

c Non‐stunted child and a nonoverweight mother pair defined as child having a height‐for‐age *Z*‐score (HAZ) ≥ −2 *SD* above median and a mother with a BMI <25.

d Stunted child and a nonoverweight mother pair defined as child having a height‐for‐age *Z*‐score (HAZ) < −2 SD below median and a mother with a BMI <25.

e Non‐stunted child and an overweight mother pair defined as child having a height‐for‐age *Z*‐score (HAZ) ≥ −2 SD above median and a mother with a BMI >25.

* ** ***

### 3.3 Risk factors for MCDB

When comparing nonstunted child, nonoverweight mother pairs with MCDB, the likelihood of MCDB was associated with older child age, older maternal age at child's birth, higher maternal education, lower maternal height, larger number of household members, and household practice of open defecation (Table 4). Households practicing open defecation were 3.4 times more likely to experience MCDB compared with households using safe defecation practices (RR = 3.41; 95% CI = 1.20, 9.68).

When comparing stunted child, nonoverweight mother pairs with MCDB, the likelihood of MCDB was associated with older maternal age at child's birth, higher maternal education, and lower parity. Not having a handwashing place in the house was associated with decreased odds of MCDB (OR = 0.45; 95% CI 0.21, 0.95).

When comparing nonstunted child, overweight mother pairs with MCDB, the odds of MCDB were associated with older child age, male sex, mother never married or cohabitating, lower maternal height, poorer household wealth index quintile, and larger number of household members. Mothers with shorter height either <150 cm (RR = 6.73; 95% CI = 2.44, 18.56) or between 150 and 159 cm (RR = 3.73; 95% CI = 1.40, 9.96) and mothers who were never married or cohabitating with a partner (RR = 2.32; 95% CI 1.50, 3.54) were more likely to be MCDB compared with households with an overweight mother and a nonstunted child. Households from the lowest four wealth index quintiles were more likely to be MCDB compared with the wealthiest households with the highest risk among the poorest households (RR = 4.37; 95% CI = 1.67, 11.49).

## 4 DISCUSSION

The high double burden of malnutrition in RMI is found not only at the population level but at the household level as well. Our analysis indicates that one in four households in RMI is home to an overweight mother with a stunted child. Stunting in children surpasses the WHO threshold for very high prevalence (30%), peaking during the period of complementary feeding from 6 to 23 months of age. Our analysis indicates that nearly three out of four mothers were overweight or obese, and nearly all mothers (93.7%) were shorter than 160 cm. The prevalence of child overweight (3.3%) and wasting (4.0%), however, was low, and few mothers (2%) were underweight.

Perhaps due to the very high prevalence of maternal overweight in RMI, the main drivers of child stunting were similar to those for MCDB. The risks of child stunting and of MCDB compared with nonstunted children and nonstunted child overweight mother pairs were largely associated with maternal characteristics and household economic status. The higher likelihood of stunting and MCDB among children of shorter mothers suggests adverse intergenerational nutritional impacts on birth size and growth limiting the attainment of genetic height potential (De Onis & Branca, 2016). These findings are consistent with results from a meta‐analysis of 109 DHS country surveys that showed an inverse association between maternal height and the prevalence of child stunting (Ozaltin et al., 2010). The steep increase in stunting between 6 and 18 months of age likely reflects the cumulative effect of growth faltering in utero and early childhood (WHO, 2014). The associations between low household wealth and large household size with child stunting and MCDB may be indicative of economic barriers to achieving adequate child nutrition and improved household living environments. Poor household living environments were predictors of child stunting but were associated with MCDB. Practice of open defecation was associated with 3.4 times increased risk of MCDB compared with nonstunted child nonoverweight mother pairs with similar wealth index status. Not having a place for handwashing in the house was protective of MCDB compared with stunted child nonoverweight mother pairs. Further exploration of how household living environments influence child stunting and MCDB is needed. It is noteworthy that mothers who were never married or were not cohabitating with a partner at the time of the survey—nearly half of the mothers in our sample—had increased odds of having a stunted child or being MCDB. This finding indicates that single mothers may have additional limitations on economic resources and parental care, which require further exploration.

As the prevalence of maternal overweight and obesity in the survey was very high, MCDB pairs were markedly different from households with a stunted child/nonoverweight mother pair or nonstunted child/nonoverweight mother pairs. Compared with pairs with nonoverweight mothers, increased risk of MCDB was associated with older maternal age, attainment of higher education, and compared with stunted child/nonoverweight mother pairs, lower parity. These factors may reflect the risk of maternal overweight rather than child stunting with older, higher education mothers more likely to be overweight regardless of having a stunted child. In comparison to nonstunted/overweight mother pairs, however, MCDB pairs are significantly poorer.

Child stunting, regardless of the nutrition status of the mother, is influenced by shorter maternal height and indicators of poverty in RMI. Risk of MCDB, where the child is stunted and the mother is overweight, increases with maternal age and higher education and remains associated with poorer households. Although not measured in this study, the high prevalence of MCDB in RMI is likely linked with the nutrition transition in the country. Previous research has shown that low consumption of nutrient‐rich foods and high consumption of nutrient‐poor foods by women and children are strongly associated with both an increased risk of stunting in children (Arimond & Ruel, 2004; Ihab et al., 2014; Rah et al., 2010; Sawadogo et al., 2006) and a higher risk of overweight and obesity in their mothers (Aitsi‐Selmi, 2015).

Globally, the coexistence of maternal overweight and child stunting (MCDB) is linked to early life undernutrition exacerbated by the nutrition transition (Delisle, 2008; Popkin, Adair, & Ng, 2012; Gillespie & Haddad, 2001). In a context of nutrition transition, children stunted during the first 1,000 days are likely to undergo metabolic and physiological changes in response to undernourishment that increase their susceptibility to becoming overweight and developing related NCDs later in childhood, adolescence, and adulthood (Hoffman, Roberts, et al., 2000; Hoffman, Sawaya, et al., 2000; Sawaya, 2003; Sawaya et al., 1998). As children who are stunted after 2 years of age have missed the critical window for optimal growth, the high burden of early stunting in RMI has broader implications for the health and economic capacity of the entire population.

There are a few limitations to our analysis. We investigated the effects of various household, maternal, and child factors on child stunting and MCDB using a cross‐sectional study design, which prevents the inference of causal relationships. Additionally, an insufficient sample size precluded a representative analysis of the effects of low birthweight and infant and young child feeding practices on the risk of stunting as these indicators were collected in subsamples of the total population. Further research should determine the effects of these important indicators on child stunting in the RMI context.

## 5 CONCLUSION

The public health action in RMI has focused on the prevention and treatment of overweight, obesity, and NCDs in adolescents and adults. However, the high prevalence of child stunting and MCDB found in our analysis represents a major public health challenge for the country. Preventing childhood stunting is vital for ensuring children's physical and cognitive development, preventing future overweight, NCDs, and associated consequences in later life, and for achieving greater social and economic gains for the country. Universal and targeted measures are recommended to address the driving factors to child stunting and maternal overweight, which include addressing poor maternal nutrition during pregnancy and increasing access, affordability, and demand to nutrient‐rich foods for young children and their mothers, especially among the most vulnerable social groups. The findings of high double burden of malnutrition in RMI 2017 have brought about high‐level commitment by the nation's president to mobilize resources for promoting services and support to mothers and children during the 1,000 days from conception to age 2 years. Continued high‐level advocacy with integrated and synergistic action across programmes is needed to reduce the double burden of stunting and overweight among children and women in RMI.

## CONFLICTS OF INTEREST

The authors declare that they have no conflicts of interest.

## CONTRIBUTIONS

The authors' responsibilities were as follows—JLB and SG designed the study; JLB acquired and analysed data; JLB, SG, UP, and WE interpreted the data; JLB drafted this manuscript; and JB, SG, UP, JA, FdB, and WE provided critical intellectual feedback to help revise the manuscript. All authors have read and approved the final manuscript.

## Footnotes

*
*p* < .05.

**
*p* < .01.

***
*p* < .001.

## References

[mcn12832-bib-0001] Aitsi‐Selmi, A. (2015). Households with a stunted child and obese mother: Trends and child feeding practices in a middle‐income country, 1992‐2008. Maternal and Child Health Journal, 19, 1284–1291. 10.1007/s10995-014-1634-5 25500760 PMC4445768

[mcn12832-bib-0002] Arimond, M. , & Ruel, M. T. (2004, October 1). Dietary Diversity is associated with child nutritional status: Evidence from 11 Demographic and Health Surveys. The Journal of Nutrition, 134(10), 2579–2585. 10.1093/jn/134.10.2579 15465751

[mcn12832-bib-0037] Bosy Westphal A., P. D. (2009). Short stature and obesity: positive association in adults but inverse association in children and adolescents. The British Journal of Nutrition, 102(3), 453–461. 19250584 10.1017/S0007114508190304

[mcn12832-bib-0003] De Onis, M. , & Branca, F. (2016). Childhood stunting: A global perspective. Matenral and Child Nutrition, 12(Suppl 1), 12–26. 10.1111/mcn.12231 PMC5084763 27187907

[mcn12832-bib-0004] Delisle, H. (2008, June). The double burden of malnutrition in mothers and the intergenerational impact. Annals of the New York Academy of Sciences, 1136(1), 172–184. 18579881 10.1196/annals.1425.026

[mcn12832-bib-0031] Doak CM., A. L. (2005). The dual burden household and the nutrition transition paradox. International Journal of Obesity, 129–136. 15505634 10.1038/sj.ijo.0802824

[mcn12832-bib-0005] Economic Policy, P. A . (2007). Republic of the Marshall Islands demographic and health survey 2007.

[mcn12832-bib-0006] Food and Agriculture Organization (FAO) (2006). The double burden of malnutrition: Case studies from six developing countries. (p. 84.) Rome: FAO Food and Nutrition Paper. 19172986

[mcn12832-bib-0041] Friedman SM, R. P. (2006). Decreased energy expenditure‐ an adaptive mechanism of nutritional growth retardation. Nutrition Research, 26, 345–349.

[mcn12832-bib-0040] Frisancho, A. (2003). Reduced rate of fat oxidation: a metabolic pathway to obesity in the developing nations. American Journal of Human Biology, 15, 5220532. 10.1002/ajhb.10191 12820194

[mcn12832-bib-0007] Fry, K. , Firestone, R. , Chakraborty, N. M. (2014). Measuring equity with nationally representative wealth quintiles. Washington (DC): Population Services International; 2014

[mcn12832-bib-0035] Garrett J. L., R. M. (2005). The coexistence of child undernutrition and maternal overweight: Prevalence, hypotheses, and programme and policy implications. Maternal and Child Nutrition, 26(2), 1 (3), 185–196. 10.1111/j.1740-8709.2005.00034.x PMC6860962 16881899

[mcn12832-bib-0008] Ghattas, H. (2014). Food security and nutrition in the context of the nutritino transition. *Technical Paper*.

[mcn12832-bib-1009] Gillespie, S. , & Haddad, L. (2001). *Attacking the double burden of malnutrition in* *Asia and the Pacific* . ADB Nutrition and Development Series Number 4.

[mcn12832-bib-0042] Grillo LP., S. A. (2005). Lower resting metabolic rate and higher velocity of weight gain in a prospective study of stunted vs non stunted girls living in the shanty towns of Sao Paulo, Brazil. European Journal of Clinical Nutrition, 59, 835–842. 15900308 10.1038/sj.ejcn.1602150

[mcn12832-bib-0034] Gubert MB., S. A. (2016). Understanding the double burden of malnutrition in food insecure households in Brazil. Maternal and Child Nutrition. 10.1111/mcn.12347 PMC6866242 27502214

[mcn12832-bib-0009] Helble, M. , & Francisco, K. (2017). The upcoming obesity crisis in Asia and the Pacific: First Cost Estimates. Tokyo: Asian Development Bank Institute: ADBI Working Paper 743.

[mcn12832-bib-0010] Hoffman, D. (2001). Obesity in developing countires: Causes and implications.

[mcn12832-bib-0011] Hoffman, D. , Roberts, S. , Verreschi, I. , Martins, P. , de Nascimento, C. , Tucker, K. , & Sawaya, A. (2000, September 1). Regulation of energy intake may be imparied in nutritionally stunted children from shantytowns of Sao Paulo, Brazil. Journal of Nutrition, 130(9), 2265–2270. 10.1093/jn/130.9.2265 10958822

[mcn12832-bib-0012] Hoffman, D. J. , Sawaya, A. L. , Coward, W. A. , Wright, A. , Martins, P. A. , de Nascimento, C. , … Roberts, S. B. (2000). Energy expenditure of stunted and non stunted boys and girls living in the shantytowns of Sao Paulo, Brazil. The American Journal of Clinical Nutrition, 72(4), 1025–1031. 10.1093/ajcn/72.4.1025 11010947

[mcn12832-bib-0013] Ihab, A. N. , Rohana, A. J. , Manan, W. W. , Suriati, W. W. , Zalilah, M. S. , & Rusli, A. M. (2014). The coexistence of dual form of malnutrition in a sample of rural Malaysia. International Journal of Preventive Medicine, 4, 690–699. PMC3733037 23930187

[mcn12832-bib-0032] Jehn M., B. A. (2009). Paradoxical malnutrition in mother child pairs: untangling the phenomenon of over and undernutrition in underdeveloped economies. Economics and Human Biology, 28–25. 19246260 10.1016/j.ehb.2009.01.007

[mcn12832-bib-0014] Kosaka, S. , & Umezaki, M. (2017). A systematic review of the prevalence and predicators of the double burden of malnutrition within households. British Journal of Nutrition, 117, 1118–1127. 10.1017/S0007114517000812 28514987

[mcn12832-bib-0033] Lee J., H. R. (2010). Disentangling nutritional factors and household characteristics related to child stunting and maternal overweight in Guatemala. Economics and Human Biology, 8, 188–196. 20541480 10.1016/j.ehb.2010.05.014

[mcn12832-bib-0039] Oddo VM., J. H. (2012). Predictors of maternal and child double burden of malnutrition in rural Indonesia and Bangladesh. American Journal of Clinical Nutrition, 95(4), 951–958. 10.3945/ajcn.111.026070 22357721

[mcn12832-bib-0015] Ozaltin, E. , Hill, K. , & Subramanian, S. V. (2010). Association of maternal stature with offspring mortality, underweight, and stunting in low‐ to middle‐income countries. JAMA, 303, 1507–1516. 10.1001/jama.2010.450 20407060 PMC3100588

[mcn12832-bib-0016] Popkin, B. (2002, March). The shift in stages of the nutriton transition in the developing world differs from past experiences! Public Health Nutrition, 5(1A), 205–214. 10.1079/PHN2001295 12027286

[mcn12832-bib-0017] Popkin, B. M. , Adair, L. S. , & Ng, S. W. (2012). Now and then: The global nutrition transition, The pandemic of obesity in developing countries. Nutrition Reviews, 70(1), 3–21. 10.1111/j.1753-4887.2011.00456.x 22221213 PMC3257829

[mcn12832-bib-0018] Rah, J. H. , Akhter, N. , Semba, R. D. , De Pee, S. , Bloem, M. W. , Campbell, A. A. , … Kraemer, K. (2010). Low dietary diversity is a predictor of children stunting in rural Bangladesh. European Journal of Clinical Nutrition, 64, 1393–1398. 10.1038/ejcn.2010.171 20842167

[mcn12832-bib-0038] Ramirex‐Zea M., K.‐L. M.‐F. (2014). The double burden of malnutrition in indigenous and non indigenous Guatemalan populations. American Journal of Clinical Nutrition, 100(6), 1644S–1651S. 10.3945/ajcn.114.083857 25411307

[mcn12832-bib-0019] Republic of the Marshall Islands Ministry of Health and Human Services, R. E . (2017). *Republic of the Marshall Islands Integrated Child Health and Nutrition Survey* 2017, *Final Report* . Majuro, Republic of the Marshall Islands.

[mcn12832-bib-0020] Sawadogo, P. S. , Martin‐Prevel, Y. , Savy, M. , Kameli, Y. , Traissac, P. , Traoré, A. S. , & Delpeuch, F. (2006). An infant and child feedign index is associated with th enutritional status of 6‐to 23‐month old children in rural Burkina Faso. The Journal of Nutrition, 136, 656–663. 16484539 10.1093/jn/136.3.656

[mcn12832-bib-0021] Sawaya, A. L. (2003, May). The link between childhood undernutrition and the risk of chronic diseases in adulthood: A case study of Brazil. Nutrition Reviews, 61, 168–175. 10.1301/nr.2003.may.168-175 12822705

[mcn12832-bib-0022] Sawaya, A. L. , Grillo, L. P. , Verreschi, I. , Carlos da Silva, A. , & Roberts, S. B. (1998). Mild stunting is associated with higher susceptibility to the effects of high fat diets: Studies in a Shantytown population in Sao Paulo, Brazil. The Journal of Nutrition, 128, 415S–420S. 10.1093/jn/128.2.415S 9478039

[mcn12832-bib-0036] Sichierei R., S. C. (2003). Combined effect of short stature and socioeconomic status on body mass index and weight gain during reproductive age in Brazilian women. Brazilian Journal of Medical and Biological Research, 1319–1325. 14502363 10.1590/s0100-879x2003001000007

[mcn12832-bib-0024] Tzioumis, E. , & Adair, L. (2014, June). Childhood dual burden of under and over nutrition in low and middle income counties: A critical review. Food and Nutrition Bulletin, 35(2), 230–243. 10.1177/156482651403500210 25076771 PMC4313560

[mcn12832-bib-0025] UNICEF (2013). Improving child nutrition: The achievable imperative for global progress. New York: United Nations Children's Fund.

[mcn12832-bib-0026] WHO . (2014). Global nutrition targets 2025: Stunting policy brief. Geneva: World Health Organization (WHO/NMH/NHD/14.3).

[mcn12832-bib-0027] WHO and UNICEF . (2014). Post 2015 WASH targets and indicators: JMP.

[mcn12832-bib-0028] WHO MultiCentre Growth Reference Study Group and de Onis, M (2006). WHO Child Growth Standards based on length/height, weight and age. Acta Paediatrica. Supplement, 95, 76–85. 10.1111/j.1651-2227.2006.tb02378.x 16817681

[mcn12832-bib-0029] World Health Organization . (2016). Consideration of the evidence on childhood obesity for the Commission on Ending Childhood Obesity: Report of the ad hoc working group on science and evidence for ending childhood obesity.

[mcn12832-bib-0030] Ziaullah, M. (2014, April). Comtemporary investigations of Pakistn food insecurity and trends of global food supply and demand. IOSR Journal of Business and Management, 16(4), 54–61.

